# Involvement of NADPH Oxidase 1 in Liver Kinase B1-Mediated Effects on Tumor Angiogenesis and Growth

**DOI:** 10.3389/fonc.2018.00195

**Published:** 2018-06-04

**Authors:** Elisabetta Zulato, Francesco Ciccarese, Giorgia Nardo, Marica Pinazza, Valentina Agnusdei, Micol Silic-Benussi, Vincenzo Ciminale, Stefano Indraccolo

**Affiliations:** ^1^Istituto Oncologico Veneto IOV – IRCCS, Padova, Italy; ^2^Department of Surgery, Oncology and Gastroenterology, University of Padova, Padova, Italy

**Keywords:** NADPH oxidase 1, liver kinase B1, angiogenesis, lung cancer, reactive oxygen species, oxidative stress

## Abstract

The liver kinase B1 (*LKB1*) gene is a tumor suppressor with an established role in the control of cell metabolism and oxidative stress. However, whether dis-regulated oxidative stress promotes growth of LKB1-deficient tumors remains substantially unknown. Through *in vitro* studies, we observed that loss of LKB1 perturbed expression of several genes involved in reactive oxygen species (ROS) homeostasis. In particular, this analysis evidenced strongly up-modulated NADPH oxidase 1 (*NOX1*) transcript levels in tumor cells lacking LKB1. NOX1 accounted in part for enhanced cytotoxic effects of H_2_O_2_-induced oxidative stress in A549 LKB1-deficient tumor cells. Notably, genetic and pharmacologic inhibition of NOX1 activity reduced angiogenesis and growth of A549 tumors in mice. These results suggest that NOX1 inhibitors could counteract ROS production and the angiogenic switch in LKB1-deficient tumors.

## Introduction

Increased oxidative stress is common in cancer cells. In fact, uncontrolled proliferation is connected with profound metabolic remodeling accompanied by altered redox status. Reactive oxygen species (ROS) are important biological messengers involved in cell signaling, tumor growth, and metastasis (1). Recent studies suggest that increased oxidative stress could be exploited for therapeutic purposes (2, 3), as tumor cells might be particularly vulnerable to drugs which further increase oxidative stress or otherwise block biochemical buffers of ROS (4).

The liver kinase B1 (LKB1) is a serine threonine kinase, whose hereditable mutations are associated with the Peutz-Jeghers syndrome and is somatically mutated or deleted in certain tumor types, with relatively high frequencies in non-small cell lung cancer (NSCLC) and cervical carcinoma (5). LKB1 is involved in energy homeostasis control, as well as control of cell proliferation and polarity (6). LKB1 and its downstream effector AMP-activated protein kinase (AMPK) have also been recently involved in metabolic adaptation and in the regulation of tumor cell response to oxidative stress by various mechanisms including control of NADPH homeostasis (7) and enhancement of the activity of superoxide dismutase-2 and catalase by a p38-mediated mechanism (8). These studies largely utilized LKB1-deficient tumor cell lines and their matched LKB1-proficient counterpart to investigate effects of this kinase on oxidative stress. Although several features and mechanisms associated with altered ROS homeostasis in LKB1-mutant tumor cells have been uncovered by using this experimental system, previous studies did not analyze the transcriptional perturbations of genes involved in redox control in LKB1-deficient cell lines.

Here, we investigated at transcriptional level three pairs of LKB1-deficient cell lines and observed that LKB1 is involved in the regulation of NADPH oxidase 1 (*NOX1*) transcript expression. Inhibition of NOX1 activity attenuated cytotoxic effects of oxidative stress in LKB1-deficient tumor cells *in vitro* and was an effective strategy to prevent the angiogenic switch and growth of LKB1-deficient A549 tumors *in vivo*. Although marked heterogeneity in the expression levels of *NOX1* among LKB1-deficient lung and cervical cancer cell lines does not allow us to generalize these findings, our results hint at NOX1 as a candidate mediator of angiogenesis in a subset of LKB1-deficient tumors.

## Materials and Methods

### Cell Culture and *In Vitro* Treatments

A549 cells were provided by Dr. Reuben J. Shaw (Salk Institute for Biological Studies, La Jolla, CA, USA). H460, H1650, H1975, HeLa, SiHa, and HEK293T cells were purchased from ATCC (Manassas, VA, USA). H23, H1437, H2228, H2009, and H1299 cell lines were kindly provided by Dr. Massimo Broggini (Mario Negri Institute, Milan, Italy). HeLa, SiHa, and HEK293T cells were cultured in Dulbecco modified Eagle medium (DMEM; EuroClone, Milan, Italy), supplemented with 10% FCS (EuroClone), 10 mM HEPES, and 1% antibiotic–antimycotic mix (Thermo Fisher, Waltham, MA, USA). H460, H1650, H1975, H23, H1437, H2228, H2009, and H1299 cells were grown in RPMI1640 medium (EuroClone) supplemented with 10% FCS, 1% HEPES, 1% Na pyruvate, 2 mM l-Glutamine (Lonza, Basel, Switzerland), and 1% antibiotic–antimycotic mix. A549 cells were cultured in DMEM-F12 medium (Gibco-BRL, Grand Island, NY, USA) supplemented with 10% FCS, 2 mM l-Glutamine, and 1% antibiotic–antimycotic mix. In some experiments, cell lines were treated with the appropriate vehicle or with the following substances: 2 mM hydrogen peroxide (H_2_O_2_, Sigma-Aldrich, Saint Luis, MO, USA), 2 mM *N*-acetylcysteine (NAC, Sigma-Aldrich), 100 µM apocynin (Sigma-Aldrich), for the indicated time points.

### Retrovirus Production and Transduction With Viral Particles

The retroviral vector overexpressing human LKB1^wt^ (RV-LKB1^wt^; plasmid # 8592) and the corresponding control vector (pBABE, plasmid # 1764) used to transduce LKB1-mutated cells were purchased from Addgene (Cambridge, MA, USA). Viral particles were produced by transfecting HEK293T cells with second-generation packaging plasmids. Virus was harvested 24 and 48 h after transfection and concentrated by ultracentrifugation at 24,000 rpm for 2 h. A549, H460, and HeLa cells were transduced with a VSV-G-pseudotyped retroviral vector encoding LKB1 cDNA (LKB1^wt^) or with an empty vector (pBABE). Briefly, 0.5 × 10^6^ cells were incubated at 37°C overnight and 200 µl of concentrated vector-containing supernatant were layered over target cells. The day after, cells were washed and cultured in fresh medium. Cells expressing the constructs were selected in puromycin-containing medium (8 µg/ml for A549 and H460 cells, 4 µg/ml for HeLa cells) for 10–14 days prior to subsequent analysis.

### Reverse Transcription-PCR and Quantitative PCR

Total RNA was isolated using RNAeasy^®^ Mini Kit (Qiagen, Venlo, Netherlands) according to manufacturer’s instructions. cDNA was synthesized from 0.3 to 1 µg of total RNA using High Capacity RNA-to-cDNA Kit (Thermo Fisher). Expression levels of 20 oxidative metabolism-related genes were analyzed by real-time Ready custom panels (Roche Diagnostics, Basel, Switzerland). Real-time PCRs were performed using Platinum^®^ SYBR^®^ Green (Thermo Fisher) in an ABI Prism 7900 HT Sequence Detection System (Thermo Fisher). Results were analyzed using the ΔΔCt method with normalization against HMBS gene expression.

Genes included in Custom Panels were selected based on their role in oxidative metabolism and are reported in Table 1. Primers used for qRT-PCR are reported in Table 2.

**Table 1:** Genes included in custom panel.

Gene ID	Description	HGNC ID	Pathway
*GCLC*	*Glutamate-cysteine ligase, catalytic subunit*	4311	
*GCLM*	*Glutamate-cysteine ligase, modifier subunit*	4312	Glutathione synthesis
*GSS*	*Glutathione synthetase*	4624	

*GGT6*	*Gamma-glutamyltransferase 6*	26891	Glutathione degradation
*GGT7*	*Gamma-glutamyltransferase 7*	4259	

*NFE2L2*	*Nuclear factor erythroid 2-like 2; nuclear respiratory factor 2*	7782	Transcription factors involved in glutathione metabolism
*JUN*	*jun proto-oncogene*	6204	

*ABCC1*	*ATP-binding cassette sub-family C member 1*	51	Glutathione adducts transporters
*ABCC2*	*ATP-binding cassette sub-family C member 2*	53	

*CAT*	*Catalase*	1516	
*TXN*	*Thioredoxin*	12435	Enzymes involved in antioxidant defenses
*SOD2*	*Superoxide dismutase-2*	11180	
*GPX1*	*Glutathione peroxidase 1*	4353	
*GSR*	*Glutathione reductase*	4623	
*MSRA*	*Methionine sulfoxide reductase A*	7377	

*NOX1*	*NADPH oxidase 1*	7889	Enzymes involved in reactive oxygen species production

*CBS*	*Cystathionine* β*-synthase*	1550	Enzymes involved in transsulfuration pathway
*CTH*	*Cystathionine* γ*-lyase*	2505	

*SLC7A11*	*Solute carrier family 7 member 11; xCT*	11059	
*SLC7A10*	*Solute carrier family 7 member 10; ASC1*	11058	Cystine and cysteine transporters

*HMBS*	*Hydroxymethylbilane synthase*	4982	
*B2M*	*Beta-2-microglobulin*	914	Reference gene
*ACTB*	*Actin, beta*	132	

**Table 2:** Primers used for qRT-PCR.

Gene ID	Forward primer	Reverse primer
*NADPH oxidase 1*	5′-GGGGTCAAACAGAGGAGAGC-3′	5′-CCACTTCCAAGACTCAGGGG-3′
*HMBS*	5′-GGCAATGCGGCTGCAA-3′	5′-GGGTACCCACGCGAATCAC-3′

### Annexin-V/Propidium Iodide Assay

Cells were incubated with Annexin-V-FITC in Hepes buffer containing propidium iodide (PI), using the Annexin-V Fluos Staining Kit (Roche Diagnostics, Mannheim, Germany). Labeled cells were analyzed on EPICS-XL cytofluorimeter using Expo32 software (Beckman Coulter, Fullerton, CA, USA). Dot plot images were obtained by using the Kalusa analysis 1.3 software (Beckman Coulter).

### Measurement of Mitochondrial Membrane Potential (Δψ)

Cells were incubated with tissue culture medium containing 15 nM tetramethylrhodamine, methyl ester (TMRM; Thermo Fisher) and 20 ng/ml Verapamil (efflux pumps inhibitor; Sigma-Aldrich) for 15 min at room temperature. Labeled cells were analyzed with an LSRII flow cytometer using the 560 nm laser and the 585/15 nm filter.

### RNAi Experiments

Cells (3 × 10^5^) were seeded in six-well tissue culture plates. The following day, Opti-MEM^®^ I Medium with GlutaMAX™ (Thermo Fisher) was mixed separately with 20 nM Stealth RNAi™ siRNA (Thermo Fisher) and with Lipofectamine^®^ RNAiMAX Transfection Reagent (Thermo Fisher) and incubated 5 min at room temperature. Separated mix were combined and, following additional 20 min of incubation, 500 µl of final mix were added, drop by drop, to adherent cells, along with 1.5 ml of culture medium. Lipofection was blocked after 6 h, through removal of medium with liposomes and addition of culture medium. Sequences of Stealth RNAi™ siRNAs are reported in Table 3.

**Table 3:** Stealth siRNAs used in transfection experiments.

Stealth siRNA name	Stealth siRNA code	Sense sequence
siRNA	Negative Control Low GC Duplex	AGCUACACUAUCGAGCAAUUAACUU
siNOX1	NOX1HSS178287	UGGGAUGAUCGUGACUCCCACUGUA

### *In Vivo* Experiments

Procedures involving animals and their care conformed to institutional guidelines that comply with national and international laws and policies (EEC Council Directive 86/609, OJ L 358, 12 December, 1987) and were authorized by the ethical committee of the University of Padova and the Italian Ministry of Health (Authorization no. 143/2012-B). For tumor establishment, both dorsolateral flanks of 8-week-old SCID mice (Charles River, Wilmington, MA, USA) were injected subcutaneously with 0.5 × 10^6^ LKB1^mut^ A549 tumor cells mixed at 4°C with liquid Matrigel (Becton-Dickinson). Tumor volume (mm^3^) was measured by caliber and calculated according to the following formula: *L* × *l*^2^ × 0.5, where *L* is the longest diameter, *l* is the shortest diameter, and 0.5 is a constant to calculate the volume of an ellipsoid. In the experiment with NAC, LKB1^mut^ A549 cells were 16 h pretreated with NAC 2 mM before injection and during tumor growth NAC was administered intraperitoneally at 150 mg/kg every day. Control mice received intraperitoneal injections of PBS. In the experiment in which expression level of *NOX1* was reduced by siRNA-mediated silencing, LKB1^mut^ A549 cells were injected 48 h after lipofection. In the experiment with apocynin, LKB1^mut^ A549 cells were 16 h pretreated with apocynin at 100 µM before injection and during tumor growth apocynin was administered *via* oral gavage at 16 mg/kg every day.

At the end of the experiment, mice were killed by cervical dislocation. For all experiments, five to six mice per group were used. The tumors were harvested by dissection and snap-frozen.

### Immunofluorescence Analysis (IFA)

Tumor vessels were labeled with a rat anti-CD31 mAb (1:50 dilution; Becton-Dickinson) followed by staining with a goat anti-rat 546 secondary antibody (Thermo Fisher). Sections of 5 µm were cut from frozen biopsies and microvessel density (MVD) was quantified by screening the CD31-stained sections for the areas of highest vascularity, as previously reported (9). Briefly, nuclei were stained with TO-PRO-3 Iodide (1:1,000 dilution; Thermo Fisher). Immunofluorescence signals were visualized on a Zeiss Axiovert 100 M confocal microscope (Carl Zeiss AG; Oberkochen, Germany), using argon (488 nm) and helium-neon (543–633 nm) laser sources. For each sample, the number of fields analyzed varied between 5 and 10 per sample, depending on the size of sections; at least 4 samples per group were analyzed. Images were collected at a magnification of 200×. MVD was quantified by screening for the areas of highest vascularity. Two independent operators blindly evaluated the images: morphologically identifiable vessels were counted as an individual vessel.

### Statistical Analysis

Results were expressed as mean value ± SD. Student’s *t*-test or Mann–Whitney test were used depending on the distribution of values. Differences were considered statistically significant when *P* < 0.05. For each cell line, data are expressed as fold change arbitrarily setting controls at 1. If not specified otherwise, the symbol ^§^ was used for comparison between LKB1^mut^ and LKB1^wt^ cells, the symbol * for comparison between treated and untreated cells and the symbol ^#^ for comparison between treated cells and cells pre-treated with other drugs (NAC, Apocynin) or siRNA.

## Results

### LKB1 Regulates Expression of Genes Involved in ROS Metabolism in Tumor Cells

**Figure 1:**
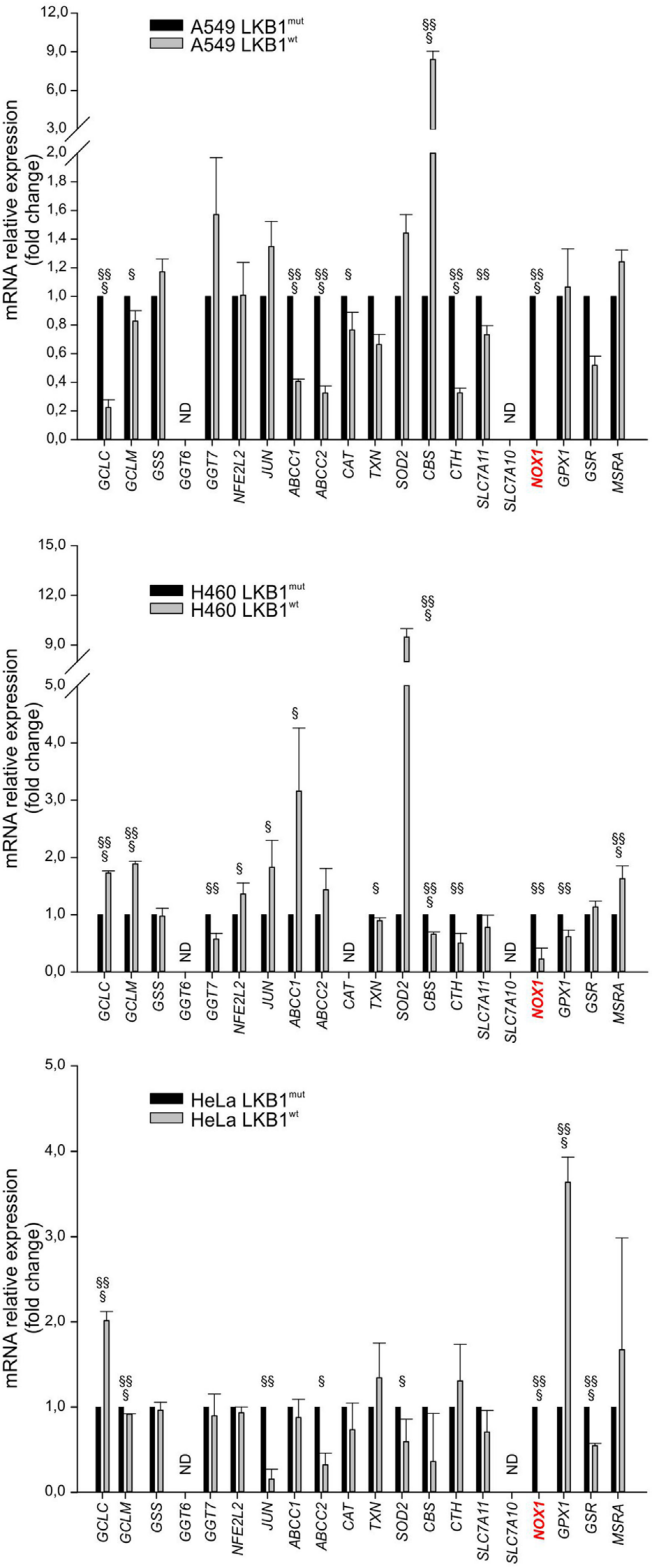
Liver kinase B1 (LKB1) *status* affects expression of oxidative metabolism-related genes. Expression profile of selected genes in isogenic pairs of A549 (top), H460 (middle), and HeLa (bottom) cells. Transcription levels of each gene were quantified by real-time Ready Custom Panels and normalized to *HMBS* mRNA content. Results are reported as mean value ± SD of three independent experiments and are expressed as fold change with respect to LKB1^mut^ cells, arbitrary set as 1 for each analyzed transcript (^§^*P* < 0.05, ^§§^*P* < 0.01, ^§§§^*P* < 0.001). ND = expression level not determined.

To investigate whether LKB1 modulated expression of genes involved in the response to cellular redox stress, we compared expression levels of 20 relevant genes (Table 1) by real-time PCR in human tumor cell lines isogenic for LKB1 *status* and grown *in vitro* under standard conditions. We performed these experiments using the LKB1-deficient NSCLC cell lines A549 and H460, and cervical carcinoma cell line HeLa transduced with a control retroviral vector (hereafter referred to as LKB1^mut^) and their LKB1-proficient counterparts obtained by retroviral transduction of wild-type LKB1 cDNA (hereafter referred to as LKB1^wt^). As expected, LKB1 reconstitution fully restored AMPK and acetyl-CoA carboxylase phosphorylation under glucose starvation—a condition known to trigger LKB1-dependent AMPK activation and associated with oxidative stress [(10) and data not shown]. Remarkably, in all cell lines tested, several antioxidant genes were downregulated and others were upregulated following reconstitution of LKB1 (Figure 1). Most of these variations were, however, not shared by the various cell lines analyzed. The most consistently downregulated gene in LKB1^wt^ cells was *NOX1*, a homolog of the macrophage *NOX2*, accounting for the respiratory burst in response to pathogens (11). NADPH oxidases catalyze the transfer of one electron from NADPH to oxygen, generating superoxide or H_2_O_2_, thus further increasing oxidative stress (12).

**Figure 2:**
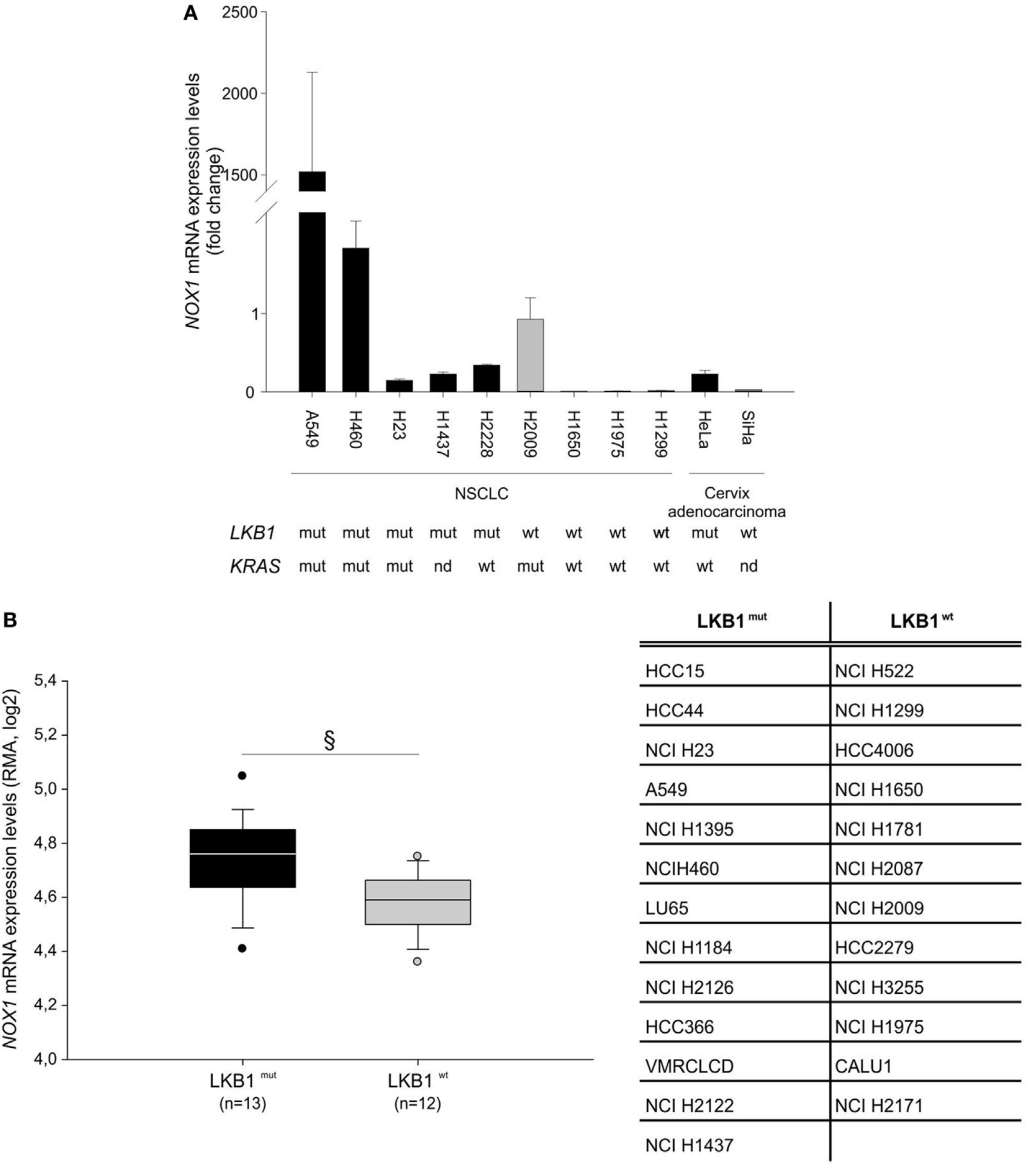
Liver kinase B1 (LKB1) controls NADPH oxidase 1 (*NOX1*) expression in human cell lines. *NOX1* expression levels in LKB1-deficient (LKB1^mut^) and LKB1-proficient (LKB1^wt^) non-small cell lung cancer (NSCLC) and cervical cancer cell lines. **(A)** Expression levels of *NOX1* mRNA in NSCLC and cervical cancer cell lines, with defined *LKB1* and *KRAS* mutational *statu*s. Results are reported as mean value ± SD of three independent experiments and are expressed as fold change compared to H2009 cells, arbitrary set at 1. **(B)** *In silico* analysis of *NOX1* levels in NSCLC cell lines. Expression level of *NOX1* as Robust Multi-array Average (RMA) values (source: Cancer Cell Line Encyclopedia). The dispersion of *NOX1* expression values in *n* = 13 *LKB1* mutated and in *n* = 12 *LKB1* wild-type cancer cell lines is represented as box-plots, indicating the first and the third quartiles and the median. The list of *LKB1*-mutated and non-mutated cancer cell lines is reported in the table (^§^*P* < 0.05). nd = mutational status not determined.

Further analysis of *NOX1* mRNA levels in nine NSCLC and two cervical cancer cell lines, with known *LKB1* mutational status, showed that almost all cell lines bearing *LKB1* mutations presented increased *NOX1* expression compared with *LKB1* wild-type cells (Figure 2A). However, broad heterogeneity in *NOX1* expression was detected in LKB1-mutant cell lines, being *NOX1* levels particularly high in A549 cells (Figure 2A). An additional *in silico* analysis was performed by matching the *LKB1* mutational status and *NOX1* mRNA transcript data of tumor cell lines, available at the COSMIC and Cancer Cell Line Encyclopedia web sites, respectively. Results showed that *NOX1* transcripts were significantly increased in LKB1-mutated NSCLC cell lines, thus strengthening our observations (Figure 2B). The relatively high expression of *NOX1* in A549 cells led us to focus on these cells to investigate the possible involvement of NOX1 in the regulation of oxidative stress.

### NOX1 Accounts for Increased Sensitivity to Exogenous Oxidative Stress in A549 Cells

**Figure 3:**
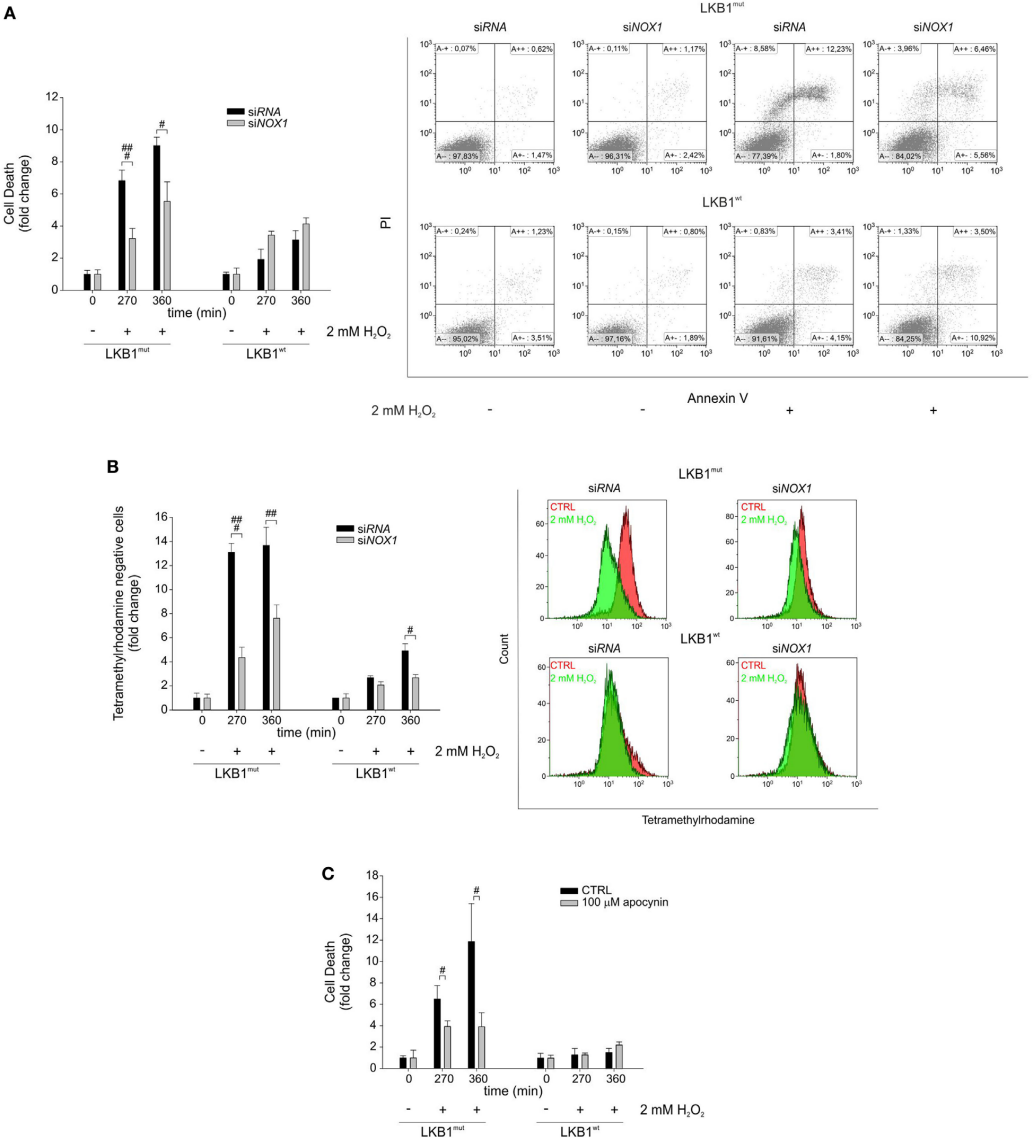
NADPH oxidase 1 (NOX1) increases sensitivity to exogenous oxidative stress *in vitro*. **(A)** A549 LKB1^mut^ and LKB1^wt^ cells were transiently transfected with si*RNA* targeting *NOX1* and cell death was measured upon H_2_O_2_ treatment (only statistically significant differences between silenced and control cells are indicated, ^#^*P* < 0.05, ^###^*P* < 0.001). A representative dot plot diagram of Annexin-V/PI stained cells is shown (right panel). **(B)** Measurement of mitochondrial membrane potential in A549 LKB1^mut^ and LKB1^wt^ cells transiently silenced for *NOX1* expression (only statistically significant differences between silenced and control cells are indicated, ^#^*P* < 0.05, ^##^*P* < 0.001, ^###^*P* < 0.001). **(C)** Induction of cell death in A549 LKB1^mut^ or LKB1^wt^ cells treated with the NOX pharmacological inhibitor apocynin 100 µM upon H_2_O_2_ treatment (only statistically significant differences between apocynin-treated cells and control cells are indicated, ^#^*P* < 0.05).

Gene silencing and pharmacologic inhibition approaches were used to analyze whether NOX1 could account for increased sensitivity to oxidative stress in LKB1^mut^ cells. Interestingly, siRNA-mediated *NOX1* silencing in A549 cells increased resistance of LKB1^mut^ A549 cells to H_2_O_2_-induced cell death, while leaving substantially unaffected response to oxidative stress in LKB1^wt^ cells (Figure 3A). Consistent with this, measurement of mitochondrial membrane potential showed a significant increase in the number of TMRM negative LKB1^mut^ A549 cells upon H_2_O_2_-treatment (Figure 3B). This phenomenon in the mitochondrial membrane potential was partially rescued by *NOX1* silencing. Similar results were obtained following NOX1 inhibition with apocynin (Figure 3C). It should be noted that despite interference with NOX1 activity, LKB1^mut^ cells showed increased H_2_O_2_-induced cell death compared with LKB1^wt^ cells (Figures 3A–C), probably due to either incomplete blockade of NOX1 or multifactorial mechanisms of control of oxidative stress by LKB1.

### NOX1 Promotes Angiogenesis and Growth of LKB1-Deficient A549 Tumors

**Figure 4:**
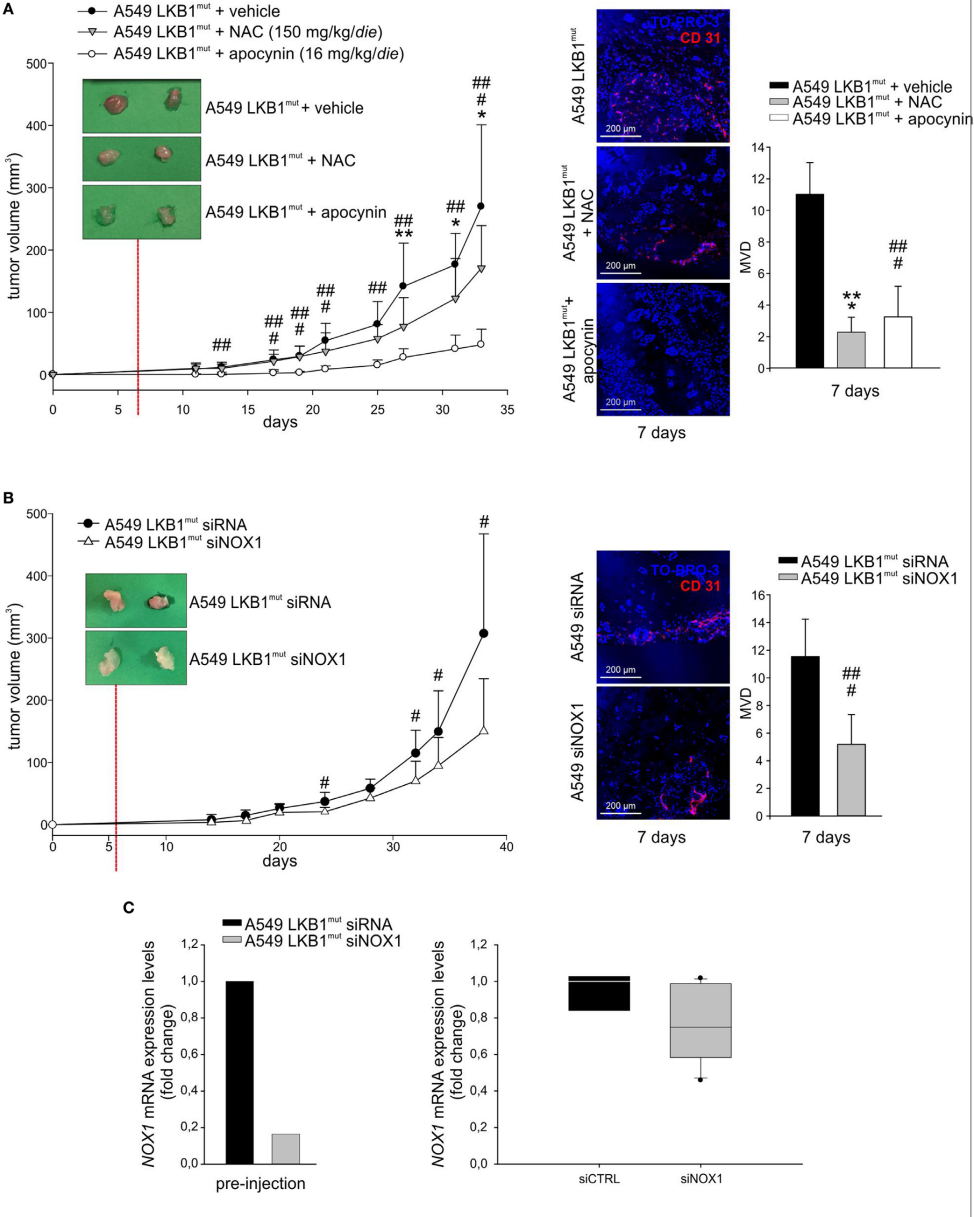
NADPH oxidase 1 (NOX1) impacts on tumor growth and angiogenesis *in vivo*. **(A)** A549 LKB1^mut^ cells were s.c. injected in SCID mice and treated daily with either *N*-acetyl cysteine (NAC) (150 mg/kg/day, gray triangles), or apocynin (16 mg/kg/day, white dots), or vehicle (black dots) (left panel). The image of two representative masses/group shows a difference in vascularization of treated compared to untreated tumors 7 days after tumor cells injection, as indicated by CD31 staining (magnification 200×) and quantification of the microvessel density (MVD) (right panel) (*n* = 3–6 fields from five samples/group; *P < 0.05, **P < 0.01, *** *P* < 0.001 A549 LKB1^mut^ NAC-treated versus untreated tumors; ^##^*P* < 0.01, ^###^*P* < 0.001 A549 LKB1^mut^ apocynin-treated versus untreated tumors). **(B)** Growth curves of s.c. tumors originated from A549 LKB1^mut^ cells transiently transfected with si*RNA* control (black dots) or with a si*RNA* targeting *NOX1* (white triangles) (left panel). Seven days after tumor cells injection, LKB1^mut^ si*RNA* tumors appear more vascularized compared to LKB1^mut^ si*NOX1* tumors, as shown by the representative image. Immunofluorescence analysis of CD31^+^ cells and quantification of microvasculature in tumors at day 7 (right panel) (*n* = 3–6 fields from five samples/group; ^#^*P* < 0.05, ^###^*P* < 0.001 A549 LKB1^mut^ si*RNA* versus si*NOX1*). **(C)** Expression levels of *NOX1* in LKB1-deficient (LKB1^mut^) cells were analyzed prior to their injection in mice (bottom panel, at left) and in samples obtained from experiment shown in B (*n* = 3 and *n* = 6 for si*RNA* and si*NOX1* samples, respectively) and recovered 1 week post injection (bottom panel, at right).

High levels of ROS have been involved in induction of angiogenesis and tumor growth (13). To investigate whether oxidative stress could play a role in LKB1^mut^ tumor formation, we co-injected LKB1^mut^ A549 cells along with administration of the antioxidant NAC. LKB1^mut^ A549 cells formed tumors (Figure 4A), and co-injection with NAC resulted in significant reduction of tumor growth and reduction of tumor vascularization, assessed 7 days after tumor cell injection (Figure 4A). Notably, chronic administration of apocynin significantly reduced tumor growth and MVD of LKB1^mut^ tumors (Figure 4A), suggesting that blockade of NOX activity impairs angiogenesis and tumor growth. These results were confirmed and strengthened by siRNA-mediated silencing of *NOX1* in A549 cells prior to their injection in mice (Figure 4B). Analysis of expression levels of *NOX1* in samples recovered 7 days after tumor cell injection showed, as expected, marginal silencing of the target gene, in contrast to the >80% reduction of *NOX1* levels at time of injection (Figure 4C). This suggests that NOX1 could play a key role in the initial phases of the angiogenic switch in the A549 LKB1^mut^ tumor xenograft model.

## Discussion

This study disclosed for the first time an effect of LKB1 on *NOX1* expression in tumor cells. According to our results, LKB1 acts as a suppressor of *NOX1* expression at the RNA level. Among a set of 20 genes involved in redox homeostasis, *NOX1* transcript was consistently down-regulated following forced expression of LKB1 cDNA in LKB1-deficient cancer cells. Other genes were also perturbed, presumably as a direct consequence of LKB1 loss or, alternatively, as adaptive response to compensate for loss of protective mechanisms. In this respect, Kaufman et al., reported that LKB1 loss leads to activation of the nuclear respiratory factor 2 transcription factor, which regulates oxidative stress response genes (14).

NADPH oxidase 1 belongs to a family of NADPH oxidase (11) and cancer cells generally express more NOX isoforms. In the case of A549 cells utilized in our study, *NOX2* was expressed at a much lower baseline level compared to *NOX1* and *NOX4* was not expressed (data not shown). Therefore, although apocynin is not an isoform-selective NOX inhibitor (11), effects measured following treatment of A549 cells with this compound are likely to be accounted for by NOX1. A second consideration regards *NOX1* levels in tumor cells. In fact, real-time PCR experiments showed marked differences in the relative expression levels of *NOX1* among the NSCLC and cervical cancer cell lines analyzed (Figure 2A). It can, therefore, be speculated that the biological consequences of NOX1 inhibition might also depend on the amount of NOX1 in tumor cells. In line with this hypothesis, we indeed observed marked reduction of H_2_O_2_-induced cell death following apocynin treatment of A549 cells but not HeLa cells, which express substantially less NOX1 transcripts (Figure 2 and data not shown). Therefore, although LKB1 acted as suppressor of *NOX1* expression in all cell lines analyzed, other endogenous factors are likely to modulate *NOX1* expression in tumor cells. According to previous studies, one of these factors could be KRAS, as its activating mutations were shown to correlate with *NOX1* overexpression in human colon cancer samples (15) and in cancer cells (16). In this regard, analysis of NSCLC cancer cell lines showed increased *NOX1* mRNA levels in cell lines bearing *LKB1* and *KRAS* mutations (Figure 2). This heterogeneity may also have precluded the inclusion of NOX1 among top ranking genes regulated by LKB1 in other genome-wide studies which previously investigated transcriptional profiles in LKB1-mutant cells and clinical samples (5, 14, 17, 18).

Although a detailed explanation of the mechanism connecting LKB1 to NOX1 is lacking and goes beyond the aim of our study, some hypothesis can be advanced. First, although LKB1 is not a transcription factor it is localized both in the cytoplasm and the nucleus of cells (17, 19). Therefore, suppression of NOX1 by LKB1 could be accounted for by its still poorly characterized nuclear functions. In this regard, it has been demonstrated that LKB1 forms a nuclear complex with the transcription regulatory factor LMO4, the transcription factor GATA-6 and Ldb1, and it has been suggested that this complex is involved in the regulation of GATA-mediated gene expression (20). Intriguingly, GATA-binding sites have been identified within the *NOX1* promoter (21). It is thus possible that LKB1 could be involved in the regulation of *NOX1* mRNA transcript through a GATA-6 mediated mechanism. Second, among indirect mechanisms, it has been shown that loss of LKB1 especially combined with KRAS mutations (as is the case in A549 cells) triggers the serine–glycine-one-carbon pathway, leading to increased S-adenosylmethionine generation, increased activity of DNA methyltransferases and elevation in DNA methylation (22). Hypothetically, this might silence some unknown repressor and thus unleash *NOX1* expression. Hence, alterations of NOX1 expression might occur as consequence of changes in epigenetic marks following vector-mediated re-expression of LKB1 in tumor cells. Further studies are required to validate these hypothesis.

The second innovative observation of this study regards the anti-angiogenic effects of drugs affecting ROS homeostasis in the context of LKB1-deficient tumors. Previous studies reported a link between angiogenesis and LKB1 expression in tumor models. Zhuang et al. described reduction of MVD accompanied by reduced expression of the angiogenic factors basic fibroblast growth factor and vascular endothelial growth factor (VEGF) in LKB1-deficient MDA-MB435 xenografts when LKB1 cDNA was added back, but further mechanistic insights were not provided (23). Along this line, it was known that NOX1 can promote the angiogenic switch in NIH-3T3 fibroblasts converting them into tumorigenic cells by a mechanism involving generation of ROS and increased expression of VEGF mRNA (24). Therefore, in view of our findings, it is conceivable that some of the effects on VEGF expression that follow interventions on LKB1 might depend on modulation of *NOX1* levels in tumor cells. Interestingly, Okon et al. reported that LKB1 functions as a RAB7 effector and suppresses angiogenesis by promoting the cellular trafficking of neuropilin-1 from the RAB7 vesicles to the lysosomes for degradation (25). Neuropilin-1 is an angiogenic receptor that can be expressed both by endothelial and tumor cells (26). When expressed by tumor cells, neuropilin-1 seems to increase VEGF-mediated angiogenesis. Therefore, it can be speculated that adding LKB1 back to tumor cells triggers complex changes in the tumor microenvironment involving NOX1, ROS, and neuropilin-1 that impact on VEGF/VEGFR-2 signaling and angiogenesis with negative rebounds on tumor growth that largely exceed the modest effects on tumor cell proliferation, which can be detected *in vitro*. As the contribution of each component of this envisioned model could vary in individual tumors, additional studies are required to validate this hypothesis both in preclinical models and in patients.

In conclusion, our study disclosed for the first time that LKB1 controls *NOX1* expression *in vitro*. Perturbing ROS homeostasis or inhibition of NOX1 reduced tumor growth and angiogenesis *in vivo* and could represent a new possible therapeutic approach in LKB1-deficient KRAS-mutated tumors.

## Ethics Statement

Procedures involving animals and their care conformed to institutional guidelines that comply with national and international laws and policies (EEC Council Directive 86/609, OJ L 358, 12 December, 1987) and were authorized by the ethical committee of the University of Padova and the Italian Ministry of Health (Authorization no. 143/2012-B).

## Author Contributions

SI conceived the study, designed experiments, analyzed data, and wrote the paper. EZ and FC designed and performed experiments, analyzed data, and wrote the paper. GN, MP, VA, and MS-B performed experiments and analyzed data. VC analyzed data and corrected the paper.

## Conflict of Interest Statement

The authors declare that the research was conducted in the absence of any commercial or financial relationships that could be construed as a potential conflict of interest.

## Abbreviations

LKB1, liver kinase B1; NOX1, NADPH oxidase 1; ROS, reactive oxygen species; NSCLC, non-small cell lung cancer; AMPK, AMP-activated protein kinase; ACC, acetyl-CoA carboxylase; CCLE, cancer cell line encyclopedia; NAC, *N*-acetyl cysteine; MVD, microvessel density; NRF2, nuclear respiratory factor 2; bFGF, basic fibroblast growth factor; VEGF, vascular endothelial growth factor; VEGFR, vascular endothelial growth factor receptor.

## References

[1] Panieri E Santoro MM. ROS homeostasis and metabolism: a dangerous liason in cancer cells. Cell Death Dis (2016) 7:e2253. 10.1038/cddis.2016.105 27277675 PMC5143371

[2] Ciccarese F Ciminale V. Escaping death: mitochondrial redox homeostasis in cancer cells. Front Oncol (2017) 7:117. 10.3389/fonc.2017.00117 28649560 PMC5465272

[3] Raza MH Siraj S Arshad A Waheed U Aldakheel F Alduraywish S ROS-modulated therapeutic approaches in cancer treatment. J Cancer Res Clin Oncol (2017) 143:1789–809. 10.1007/s00432-017-2464-9 28647857 PMC11819417

[4] Gorrini C Harris IS Mak TW. Modulation of oxidative stress as an anticancer strategy. Nat Rev Drug Discov (2013) 12:931–47. 10.1038/nrd4002 24287781

[5] Ji H Ramsey MR Hayes DN Fan C Mcnamara K Kozlowski P LKB1 modulates lung cancer differentiation and metastasis. Nature (2007) 448:807–10. 10.1038/nature06030 17676035

[6] Boudeau J Sapkota G Alessi DR. LKB1, a protein kinase regulating cell proliferation and polarity. FEBS Lett (2003) 546:159–65. 10.1016/S0014-5793(03)00642-2 12829253

[7] Jeon SM Chandel NS Hay N. AMPK regulates NADPH homeostasis to promote tumour cell survival during energy stress. Nature (2012) 485:661–5. 10.1038/nature11066 22660331 PMC3607316

[8] Xu HG Zhai YX Chen J Lu Y Wang JW Quan CS LKB1 reduces ROS-mediated cell damage via activation of p38. Oncogene (2015) 34:3848–59. 10.1038/onc.2014.315 25263448 PMC4377312

[9] Trenti A Zulato E Pasqualini L Indraccolo S Bolego C Trevisi L. Therapeutic concentrations of digitoxin inhibit endothelial focal adhesion kinase and angiogenesis induced by different growth factors. Br J Pharmacol (2017) 174:3094–106. 10.1111/bph.13944 28688145 PMC5573418

[10] Spitz DR Sim JE Ridnour LA Galoforo SS Lee YJ. Glucose deprivation-induced oxidative stress in human tumor cells. A fundamental defect in metabolism? Ann N Y Acad Sci (2000) 899:349–62. 10.1111/j.1749-6632.2000.tb06199.x 10863552

[11] Altenhofer S Radermacher KA Kleikers PW Wingler K Schmidt HH. Evolution of NADPH oxidase inhibitors: selectivity and mechanisms for target engagement. Antioxid Redox Signal (2015) 23:406–27. 10.1089/ars.2013.5814 24383718 PMC4543484

[12] Kamata T. Roles of Nox1 and other Nox isoforms in cancer development. Cancer Sci (2009) 100:1382–8. 10.1111/j.1349-7006.2009.01207.x 19493276 PMC11158854

[13] Xia C Meng Q Liu LZ Rojanasakul Y Wang XR Jiang BH. Reactive oxygen species regulate angiogenesis and tumor growth through vascular endothelial growth factor. Cancer Res (2007) 67:10823–30. 10.1158/0008-5472.CAN-07-0783 18006827

[14] Kaufman JM Amann JM Park K Arasada RR Li H Shyr Y LKB1 loss induces characteristic patterns of gene expression in human tumors associated with NRF2 activation and attenuation of PI3K-AKT. J Thorac Oncol (2014) 9:794–804. 10.1097/JTO.0000000000000173 24828662 PMC4026179

[15] Laurent E Mccoy JW III Macina RA Liu W Cheng G Robine S Nox1 is over-expressed in human colon cancers and correlates with activating mutations in K-Ras. Int J Cancer (2008) 123:100–7. 10.1002/ijc.23423 18398843 PMC3774003

[16] Park MT Kim MJ Suh Y Kim RK Kim H Lim EJ Novel signaling axis for ROS generation during K-Ras-induced cellular transformation. Cell Death Differ (2014) 21:1185–97. 10.1038/cdd.2014.34 24632950 PMC4085525

[17] Jimenez AI Fernandez P Dominguez O Dopazo A Sanchez-Cespedes M. Growth and molecular profile of lung cancer cells expressing ectopic LKB1: down-regulation of the phosphatidylinositol 3’-phosphate kinase/PTEN pathway. Cancer Res (2003) 63:1382–8. 12649203

[18] Lin-Marq N Borel C Antonarakis SE. Peutz-Jeghers LKB1 mutants fail to activate GSK-3beta, preventing it from inhibiting Wnt signaling. Mol Genet Genomics (2005) 273:184–96. 10.1007/s00438-005-1124-y 15731909

[19] Nardo G Favaro E Curtarello M Moserle L Zulato E Persano L Glycolytic phenotype and AMP kinase modify the pathologic response of tumor xenografts to VEGF neutralization. Cancer Res (2011) 71:4214–25. 10.1158/0008-5472.CAN-11-0242 21546569

[20] Setogawa T Shinozaki-Yabana S Masuda T Matsuura K Akiyama T. The tumor suppressor LKB1 induces p21 expression in collaboration with LMO4, GATA-6, and Ldb1. Biochem Biophys Res Commun (2006) 343:1186–90. 10.1016/j.bbrc.2006.03.077 16580634

[21] Adachi Y Shibai Y Mitsushita J Shang WH Hirose K Kamata T. Oncogenic Ras upregulates NADPH oxidase 1 gene expression through MEK-ERK-dependent phosphorylation of GATA-6. Oncogene (2008) 27:4921–32. 10.1038/onc.2008.133 18454176

[22] Kottakis F Nicolay BN Roumane A Karnik R Gu H Nagle JM LKB1 loss links serine metabolism to DNA methylation and tumorigenesis. Nature (2016) 539:390–5. 10.1038/nature20132 27799657 PMC5988435

[23] Zhuang ZG Di GH Shen ZZ Ding J Shao ZM. Enhanced expression of LKB1 in breast cancer cells attenuates angiogenesis, invasion, and metastatic potential. Mol Cancer Res (2006) 4:843–9. 10.1158/1541-7786.MCR-06-0118 17114342

[24] Arbiser JL Petros J Klafter R Govindajaran B Mclaughlin ER Brown LF Reactive oxygen generated by Nox1 triggers the angiogenic switch. Proc Natl Acad Sci U S A (2002) 99:715–20. 10.1073/pnas.022630199 11805326 PMC117371

[25] Okon IS Coughlan KA Zhang C Moriasi C Ding Y Song P Protein kinase LKB1 promotes RAB7-mediated neuropilin-1 degradation to inhibit angiogenesis. J Clin Invest (2014) 124:4590–602. 10.1172/JCI75371 25180605 PMC4191012

[26] Miao HQ Lee P Lin H Soker S Klagsbrun M. Neuropilin-1 expression by tumor cells promotes tumor angiogenesis and progression. FASEB J (2000) 14:2532–9. 10.1096/fj.00-0250com 11099472

